# Phytochemical profile and antioxidation activity of annona fruit and its effect on lymphoma cell proliferation

**DOI:** 10.1002/fsn3.1228

**Published:** 2019-11-30

**Authors:** Huda Mohammed Al‐Shaya, Haiwen Li, Obaid U. Beg, Anwar A. Hamama, Sarah Melissa Witiak, Paul Kaseloo, Rafat A. Siddiqui

**Affiliations:** ^1^ Food Chemistry and Nutrition Science Research Laboratory Virginia State University Petersburg Virginia; ^2^ Department of Biology College of Natural and Health Sciences Virginia State University Petersburg Virginia; ^3^ Princess Nourah Bint Abdulrahman University Riyadh Saudi Arabia; ^4^ Common Research Laboratory Agricultural Research Station College of Agriculture Virginia State University Petersburg Virginia

**Keywords:** anticancer, antioxidants, lymphoma, polyphenols, ramos‐1 cells

## Abstract

Cancers of the lymphatic system are broadly classified into Hodgkin and non‐Hodgkin types. Although lymphomas can be effectively treated with chemotherapy, this approach is associated with the risk of adverse side effects. High intake of certain vegetables and fruits is associated with a reduced risk of cancer development. We hypothesized that Annona fruit, which is rich in fibers and phytochemicals that are known to possess anticancer properties, can be effective in inhibiting lymphoma growth. The Annona fruit's fractions were extracted with water, methanol, or chloroform and then assayed for total phenolic, flavonoids, and tannins content; antioxidation activities; and inhibition of in vitro cell proliferation using the Ramos‐1 lymphoma cells. The methanol fractions contained the highest phenolics, flavonoids, and tannins content, and antioxidation activity. However, the methanol extracts of skin, pulp, and seeds had a moderate whereas the chloroform extracts of pulp and seeds had strong effects on Ramos‐1 cell proliferation. Our findings suggest that Annona fruits may be effective in the prevention or treatment of lymphoma.

## Graphical abstract

The methanol fractions of Annona fruits contained the highest phenolic content and antioxidation activity. However, the methanol extracts of skin, pulp, and seeds had a moderate whereas the chloroform extracts of pulp and seeds had strong effects on Ramos‐1 cell proliferation. Our findings suggest that Annona fruits may be effective in the prevention or treatment of lymphoma.

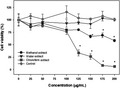

## 1 INTRODUCTION

Lymphoma is considered to be a “solid tumor” of the immune system. These malignancies originate from the cells of the immune system at different stages of their development, which leads to a very diverse range of clinical and morphologic presentations (Owens & Younes, 2016). Due to the fact that lymphatic tissue is present in many parts of the human body, lymphoma cancer can develop anywhere (Owens & Younes, 2016). Lymphomas can be broadly classified into non‐Hodgkin's lymphoma (NHL) and Hodgkin's lymphoma (HL). The exact cause of lymphoma is still not clearly understood; however, scientists have identified various causative associations. For example, the risk of NHL increases with age. In addition, NHL occurs less often in women than in men. In contrast, the risk of HL decreases during middle age, but increases during adolescence, early adulthood, and later in life (ACS, 2018). The American Cancer Society estimates that in 2018, about 83,180 new lymphoma cases were diagnosed in the United States, of which NHL will represent 74,680 cases, while HL will represent about 8,500 cases (ACS, 2018). Worldwide, NHL is the 10th most common group of cancerous diseases in women and 8th in men and it accounts for 3.0% of all cancer cases (Bray et al., 2018; Ferlay et al., 2019; Owens & Younes, 2016).

Dysregulation of the immune function is the best‐established risk factor for lymphoma (Rohrmann et al., 2007). An increased risk of lymphoma is observed in patients with autoimmune disorders. An elevated risk of developing lymphoma is observed in people who underwent organ transplantation and receive immune suppressants to prevent organ rejection (Stathis & Owens, 2016). Moreover, evidence suggests that some of the medications used in the treatment of autoimmune disorders, in particular, the tumor necrosis factor inhibitors, may also increase the risk for both NHL and HL subtypes (Calip et al., 2018). A risk for all subtypes of lymphoma is increased if an individual has a family history of lymphoma (Chang et al., 2005). In addition, environmental exposures and certain behavioral risk factors, such as excessive body weight, may also be applied to certain subtypes of lymphoma (Hartge & Smith, 2007). Another group of risk factors associated with lymphoma includes viral and bacterial infections. The Epstein–Barr virus, in particular, is associated with the following NHL subtypes: high‐grade B‐cell lymphoma, Burkitt lymphoma, and primary effusion lymphoma (Stathis & Owens, 2016). HIV is associated with the incidence of both NHL and, more rarely, HL (Hutchinson & Uner, 2007).

For most lymphomas, the standard treatment is combination chemotherapy involving doxorubicin, bleomycin, vinblastine, and/or dacarbazine (Younes, 2016); however, in certain subtypes of lymphoma cancer, radiotherapy may be used as a single treatment modality (Banerjee, 2012). Over the past decade, newly developed target agents and monoclonal antibodies have been incorporated into many lymphoma treatment approaches (Stathis, 2016).

The chemotherapies, in addition to their anticancer effect, often have a number of acute or chronic toxic side effects on cardiovascular, gastrointestinal, hematological, urogenital, respiratory, skin, renal, neurological, and reproductive systems (Hodgson, 2015).

Human epidemiological studies suggest that fruit and vegetable consumption has a protective effect on cancer of lung, stomach, rectum and colon, bladder, esophagus, breast, oral cavity and pharynx, cervix, pancreas, ovary, prostate, and endometrium (Ajila & Brar, 2012). Antioxidant compounds found in plant products, as well as a number of other naturally occurring compounds in the diet, have the potential to be used as chemopreventive agents. A list of these potential anticancer agents with known bioactive mechanisms includes flavonoids, selenium, phytosterols, dietary fiber, isothiocyanates, isoflavones, vitamin E, vitamin C, folic acid, carotenoids, and indoles (Ajila & Brar, 2012). Annona is consumed as fruits, but pulp, seeds, leaves, and bark are also widely used in folk medicine (Formagio et al., 2013). Studies have shown that phytoconstituents and peptides obtained from various parts of Annona plants have potential antimicrobial and cytotoxic properties (Jamkhande, Ajgunde, & Jadge, 2017; Wélé et al., 2004). In particular, flavonoids present in seeds, stems, bark, roots, and fruits of Annona are considered as promising chemopreventive agents, while Annonaceous acetogenins isolated from the seeds of Annona show potent cytotoxicity against many cancer cell lines (Coria‐Téllez, Montalvo‐Gónzalez, Yahia, & Obledo‐Vazquez, 2018). In addition, a cytotoxic heptapeptide was isolated from seeds of *Annona cherimola* that caused cell death in the KB cell line (Wélé et al., 2004). Based on the rationale that different parts of Annona fruit contain variable amounts of polyphenols, we hypothesize that Annona fruit, which is rich in fibers and phytochemicals, can be very effective in inhibiting lymphoma cancer growth. The objective of this study was to characterize phytochemical content and antioxidation activity of different parts of Annona fruits and to test these fractions for their effect on lymphoma cell proliferation in vitro.

## 2 MATERIALS AND METHODS

### 2.1 Materials

Cherimoya (*Annona cherimola)* fruits were purchased from a local market. Ramos‐1 cells (CRL‐1596) were purchased from ATCC (20,110). RPM 1,640 media was from Gibco. Fetal bovine serum (FBS‐BBT) was from RAMBIO, phosphate‐buffered saline streptomycin and penicillin antibiotics were from Fisher. Premixed WST‐1 Cell Proliferation Assay System was purchased from Takara BIO INC (Kusatsu). The remaining reagents required for experiments, including trolox, quercetin, catechin, tannic acid, Folin–Ciocalteu reagent, gallic acid, and diphenyl‐1‐picrylhydrazyl (DPPH) were purchased from Sigma Chemical Co. (St. Louis).

### 2.2 Isolation of annona fractions

The *Annona cherimola* fruits were washed thoroughly with distilled water and then dried with a paper towel. The skin of the fruit was removed with a kitchen peeler, and the skin peels were scraped to remove any remaining deposits of pulp, followed by washing with distilled water, which was done to remove the remaining juice. The skin, seeds, and pulp of Cherimoya were cut into pieces of about 1 cm^3^ each and then freeze‐dried. The dried fractions were ground to a fine powder using a mortar and pestle and the dried powders, after flushing with nitrogen, were stored at −80°C until used.

### 2.3 Preparation of annona extract

The methanol and water extracts of Annona fruits were prepared by mixing 5 g of dried pulp, seeds or skin powder with 200 ml of either 80% methanol or water and by placing the resulting mixtures on a shaker at room temperature overnight. The following day, the mixtures were centrifuged (20 min, 3,500*g*) using a Thermo Scientific centrifuge. The supernatant was collected, after which the residues were washed twice by repeating the steps previously performed. The resulting residues were discarded, while the collected supernatants were pooled together. The chloroform extracts were prepared according to the Folch method (Folch, Lees, & Stanley, 1957). Briefly, 1 g of dried skin, pulp, or seed powder was mixed with 20 ml of chloroform‐methanol mixture (2:1) and then 4 ml of PBS was added to this mixture. The solution was mixed on a shaker for 2 hr at room temperature. Finally, the solution was centrifuged at 3,500*g* for 20 min to separate the chloroform and water layers. The lower chloroform layer containing water‐insoluble compounds was carefully removed. The water extracts were dried using a freeze dryer, whereas a nitrogen evaporator (Organomation Associates, Inc) was used to dry the methanol and chloroform extracts. The prepared extracts were then stored in a freezer at −20ºC.

### 2.4 Preparation of stock annona solutions

The dried water, methanol, and chloroform extracts of seeds, pulp, and skin were dissolved in dimethyl sulfoxide (DMSO) to prepare a stock solution. The concentration of the stock solution was 250 µg/ml.

### 2.5 Characterization of annona extracts

*Annona cherimola* extracts were used to determine total phenolics, flavonoids, tannins content, total antioxidation activity (FRAP assay), and oxygen scavenging activity (DPPH assay).

*Total phenolic content*: The Folin–Ciocalteu method was used to determine the total phenolic content of studied *Annona cherimola* extracts (Yu et al., 2002) using gallic acid as a standard. All data are expressed as μg gallic acid equivalents/mg of dry powder (μg GA/mg dry powder).

*Flavonoid content*: Flavonoid represents a diverse class of compounds and their determination is often based on the use of appropriate standard and spectophotometric procedure involving the reaction of AlCl3 in the presence or absence of sodium Nitrite (NaNO_2_). We employed two different procedures to determine flavonoid as described (Pekal & Pyrzynska, 2014; Struchkov, Beloborodov, Kolkhir, Voskoboynikova, & Savvateev, 2018). Briefly, Procedure I involved reaction with AlCl_3_ without NaNO_2_ using quercetin dihydrate as a standard whereas in Procedure II samples were nitrozalide with NaNO_2_ before adding ALCl_3_ and catechin was used as a standard. Samples from both procedures were scanned at 300 to 600 nm. Procedure I showed optimal absorption at 425 whereas Procedure II exhibited optimal absorption at 330. The data are expressed as either μg quercetin equivalent/mg of dry powder (μg QER/mg dry powder) or μg catechin equivalent/mg dry powder (μg CTC/mg dry powder).

*Total Tannin Content:* The Folin–Ciocalteu method was used to determine the total phenolic content of studied *Annona cherimola* extracts using tannic acid as a standard (Afify, El‐Beltagi, El‐Salam, & Omran, 2012). All data are expressed as μg tannic acid equivalents/mg of dry powder (μg TNA/mg dry powder).

*FRAP Assay*: A modified version of the ferric reducing antioxidant potential (FRAP) assay was performed to determine the ferric reducing power of the studied Annona extracts (Benzie & Strain, 1996) using FeCl_3_ as a standard. Data are expressed as μM of FeCl_3_ equivalent/g dry powder).

*DPPH Assay*: The DPPH assay was used to determine the antioxidation activity of the studied Annona extracts (Cheng, Moore, & Yu, 2006) using Trolox as standard. Data are expressed as mM Trolox equivalent/g dry powder.

### 2.6 Cell culture of lymphoma cells

The lymphoma Ramos‐1 cells (CRL‐1596) were cultured in Roswell Park Memorial Institute (RPMI) media supplemented with antibiotics penicillin and streptomycin (1%) and fetal bovine serum (10%). Cells were incubated in 75 mm^2^ flasks in a humidified incubator at 37°C in the presence of 5% CO_2_ for routine culturing.

### 2.7 Cell proliferation assay

Cells were subcultured in a 96‐well plate (10,000 cells/well) by placing them in a CO_2_ incubator for 24 hr at 37°C. The cells were then treated with extracts of Annona fractions at varying concentrations (0, 10, 25, 50, 75, 100, 150, 200, and 300 µg/ml) in a serum‐free media for another 24 hr. The extent of cell proliferation was determined by the addition of WST‐1 cell proliferation reagent (20 µl) in each well and further incubating the 96‐well plate in a CO_2_ incubator for 3 hr at 37°C. The change in WST‐1 dye color due to reduction from mitochondrial dehydrogenases was recorded at 420 nm in a 96‐well plate reader.

### 2.8 Fatty acid analysis

Fatty acid composition in the skin, pulp, and seed extracts was determined using gas chromatography as described previously (Bhardwaj, Hamama, Rangappa, Joshi, & Sapra, 2003). Briefly, fatty acid methyl esters (FAMEs) were prepared by an acid‐catalyzed transesterification method. The samples (5 mg) were vortexed with 2 mLof sulfuric acid: methanol (1:99 v/v) in 10‐ml glass vials containing a Teflon boiling chip. The open vials were placed in a heating block at 90°C until the sample volume was reduced to 0.5 ml. After cooling to room temperature, 1 ml of hexane followed by 1 ml of distilled water were added. The mixture was vortexed and the upper hexane layer containing the FAME was taken and dried over anhydrous Na_2_SO_4_. The hexane phase containing FAME was transferred to a suitable vial and kept under N_2_ at 0°C for gas chromatographic analysis. Analyses of FAME were carried out using a SupelcoWax 10 capillary column (25 m × 0.25 mm L × i.d. and 0.25 μm film thickness (SupelcoWax, Inc.) in a gas chromatograph equipped with a flame ionization detector (FID) (model Vista 6,000; Varian). An integrator (SP‐4290; Spectra Physics) was used to determine relative concentrations of the detected fatty acids. Peaks were identified by reference to the retention of FAME standards and quantified by the aid of heptadecanoic acid (17:0) as an internal standard. The concentration of each fatty acid was calculated as a percentage (w/w) of the total fatty acids.

### 2.9 Data analysis

The data are expressed as the mean ± standard deviation (*SD*) for 3 or more replicates. The data comparisons were made by either unpaired Student's *t* test or one‐way ANOVA with the Tukey HSD post hoc test using SPSS software. Data with different symbols (letters) represent a significant difference within groups or with an “*” represent a significant difference between control and treated cells with *p* < .05.

## 3 RESULTS

### 3.1 Characterization of annona extracts

Our initial analysis found (data not shown) that the pulp of the fruit contributes 72.94% of the whole Annona fruit mass whereas skin and seeds occupied only 13.96% and 13.10%, respectively. Furthermore, the pulp and skin were very hydrated and contained 78.95% and 74.18% water, respectively, whereas the seeds contained only 30.46% of water. We also determined the relative amount of extractable material in different solvents. The percent extract recovered was almost same (3%–4%) in the water and methanol extracts of both skin and pulp, but was very low in seeds (~1%); however, more material was extracted from skin (9%) and seeds (24%) in chloroform.

### 3.2 Determination of phytochemicals contents in annona fruit

**Figure 1:**
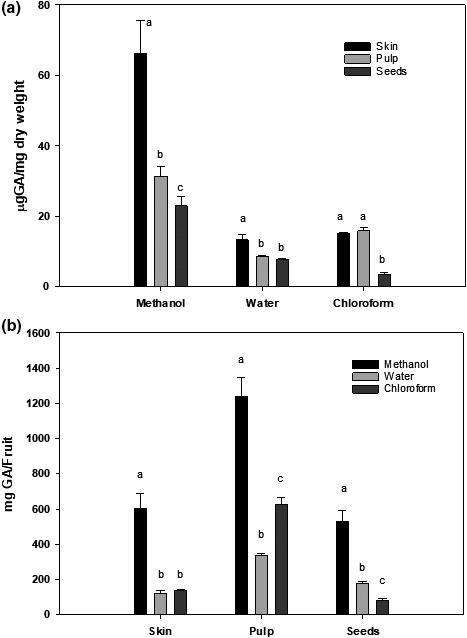
Total polyphenolic content in different fractions of Annona fruit. The polyphenolic contents in methanol, water, and chloroform extracts of skin, pulp, and seeds of Annona fruit were determine by a Folin–Ciocalteu method as described in the text. The data are reported as (a) µg GA/mg dry weight or (b) mg GA/fruit and expressed as mean ± *SD* for at least 3 individual experiments. The data were analyzed by one‐way ANOVA with the Tukey HSD post hoc test. Data labeled with different symbols represent a significant difference within groups at *p* < .05

As shown in Figure 1a, the total contents of polyphenols in the skin fraction of Annona fruit were 66.30 ± 9.23 μg GA/mg dry weight (mean ± *SD*) weight of polyphenols in the methanolic extract and were 13.33 ± 1.58 and 15.11 ± 0.38 μg GA/mg dry weight in the water and chloroform extracts, respectively. The total contents of polyphenols in the pulp fraction of Annona fruit contained 31.43 ± 2.79 μg GA/g dry weight in methanol extract, 15.83 ± 1.07 μg/mg dry weight in the chloroform extract, and only 8.89 ± 0.24 μg GA/mg dry weight in the water extract.

The phenolic contents in seeds were about 22.93 ± 2.58 μg GA/mg dry weight in the methanol fraction whereas the water extract contained about 7.64 ± 0.42 μg GA/mg dry weight and the chloroform extract contained the lowest amount of phenolic compounds (3.50 ± 0.58 μg GA/mg dry weight). The data in Figure 1a was replotted and expressed as the total amounts of phenolic compounds in an average Annona fruit (Figure 1b). Although the methanol extract of skin has the highest concentration of phenolic compounds (0.6g/fruit), the pulp contained the highest total amounts of polyphenols (1.3g/fruit), as it contained about 75% of the total fruit weight.

**Figure 2:**
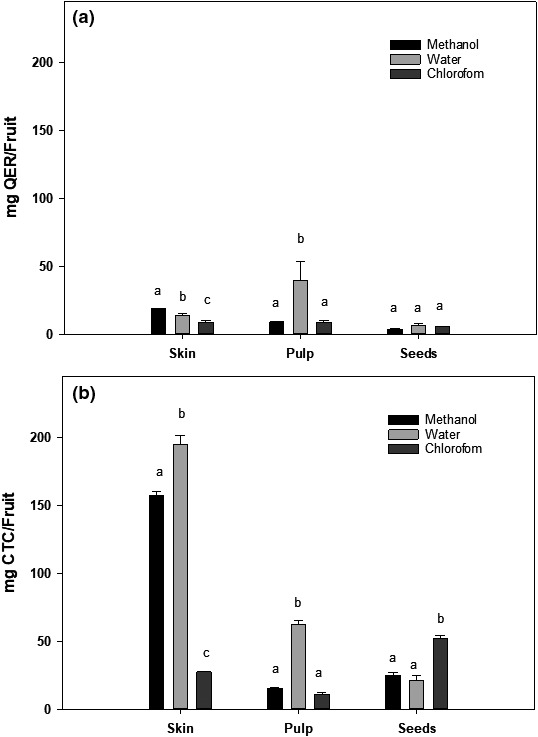
Flavonoid content in Annona Fruit. The flavonoid content in methanol, water, and chloroform extracts of skin, pulp, and seeds of Annona fruit were determine as described in the text by reaction with AlCl_3_ (a) in the absence of NaNO_3_ using quercetin (QER) as a standard or (b) in the presence of NaNO_2_ using catechin (CTC) as a standard. The data are reported as mg quercetin (QER) or catechine (CTC)/fruit and expressed as mean ± *SD* for at least 3 individual experiments. The data were analyzed by one‐way ANOVA with the Tukey HSD post hoc test. Data labeled with different symbols represent a significant difference within groups at *p* < .05

Data in Table 1 show a comparison of flavonoid content in different fractions of Annona fruit as determined using two different methods involving no‐nitrozilation or nitrozilation of samples before reaction with AlCl_3_. It is clear from data that a higher concentration of flavonoids was detected when both methanol and chloroform fractions were reacted with NaNO_2_ and AlCl_3_ ;however, the water extract of all fractions yielded similar flavonoid contents by both methods. The highest concentration of flavonoids was present in methanolic extract of skin (2.07 ± 0.018 μg QER/mg dry powder; 17.25 ± 0.34 μg CTC/mg dry powder) whereas pulp methanol fraction also contained a substantial amount of flavonoids (4.96 ± 0.16 μg CTC/mg dry powder). The water and chloroform extracts of skin, pulp, and seeds contained a very low concentration of flavonoids (less than 2.5 μg QCR or CTC/mg dry powder). The flavonoid content in whole fruit is shown in Figure 2. Both methanol and water extracts of total skin contained higher concentrations of flavonoids than pulp and seeds (150–200 μg QCR or CTC/mg dry powder).

**Table 1:** Flavonoid in annona fruits fractions

Fraction	Extracts	Procedure I ‐NaNO_3_/Quercetin (µg QER/mg Dry Powder)	Procedure II +NaNO_3_/Catechin (µg CTC/mg Dry Powder)
Skin	Methanol	2.08 ± 0.02	17.25 ± 0.34^*^
	Water	1.02 ± 0.05	1.70 ± 0.06
	Chloroform	0.40 ± 0.03	2.76 ± 0.23^*^
Pulp	Methanol	0.36 ± 0.02	4.96 ± 0.16^*^
	Water	1.00 ± 0.35	1.58 ± 0.09
	Chloroform	0.15 ± 0.06	0.55 ± 0.08^*^
Seeds	Methanol	0.28 ± 0.07	1.19 ± 0.01^*^
	Water	0.39 ± 0.04	0.47 ± 0.06
	Chloroform	0.25 ± 0.01	2.27 ± 0.09^*^

Note

Values are reported as Mean ± *SD* of three experiments. Data were analyzed using an unpaired Student's *t* test. Means in a column with “*” are significantly different between procedures I and II at *p *<* *.05.

**Figure 3:**
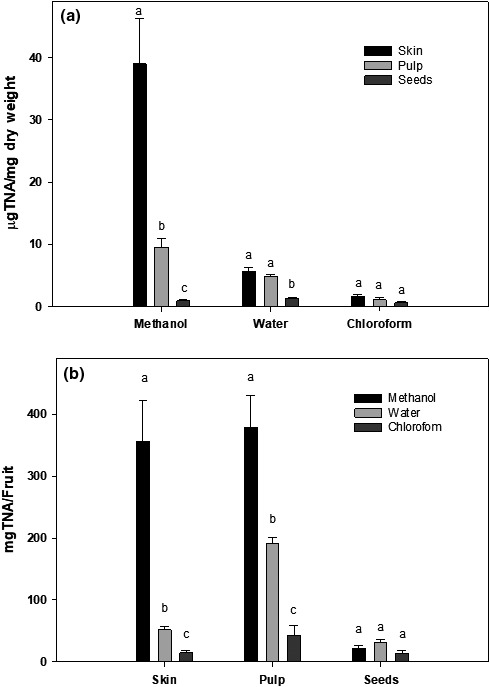
Total tannin content in different fractions of Annona fruit. The total tannin content in methanol, water, and chloroform extracts of skin, pulp, and seeds of Annona fruit were determined by a Folin–Ciocalteu method as described in the text. The data are reported as (a) µg tannic acid (TAE)/mg dry weight or (b) mg TEA/fruit and expressed as mean ± *SD* for at least 3 individual experiments. The data were analyzed by one‐way ANOVA with the Tukey HSD post hoc test. Data labeled with different symbols represent a significant difference within groups at *p* < .05

The total tannin contents in Annona fruits are shown in Figure 3. The total tannin contents in the skin fraction of Annona fruit were 38.97 ± 7.23 μg TNA/mg dry weight in the methanolic extract and 5.69 ± 0.54 and 1.62 ± 0.40 μg TNA/mg dry weight in the water and chloroform extracts, respectively. The total tannin contents of the pulp fraction of Annona fruit were 9.58 ± 1.31 μg TNA/g dry weight in methanol extract, 4.85 ± 0.25 μg TNA/mg dry weight in the chloroform extract, and only 1.09 ± 0.40 μg TNA/mg dry weight in the water extract.

The tannin contents in seeds were about 0.95 ± 0.17 μg TNA/mg dry weight in the methanol fraction whereas the water extract contained about 1.35 ± 0.18 μg TNA/mg dry weight and the chloroform extract contained the lowest amount of phenolic compounds (0.61 ± 0.19 μg TNA/mg dry weight). The data in Figure 3a were replotted and expressed as the total amounts of phenolic compounds in an average Annona fruit (Figure 3b). Although the methanol extract of skin has the highest concentration of tannin compounds, both skin and pulp contained the highest total amounts of tannins (350 –380 g/fruit).

### 3.3 Determination of antioxidation potential of annona fruits fractions

**Figure 4:**
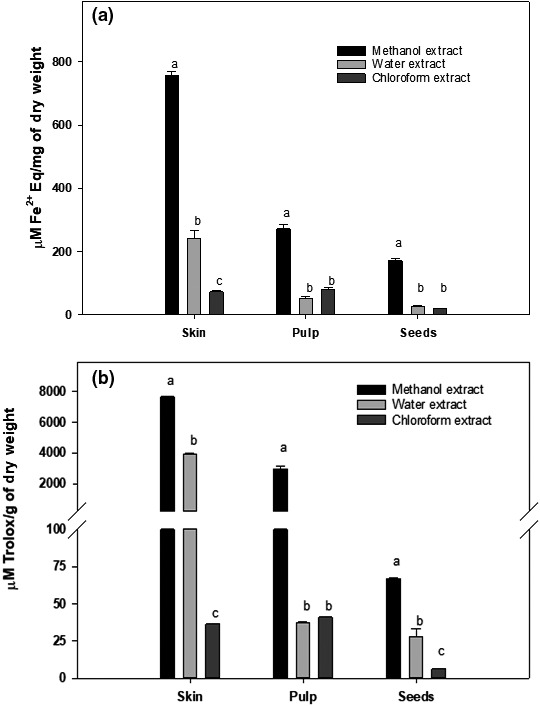
Antioxidation activity in different fraction of Annona fruits. The antioxidation activity in methanol, water, and chloroform extracts of skin, pulp, and seeds of Annona fruit were determined by (a) FRAP method using FeCl3 as a standard and (b) DPPH using Trolox as a standard as described in the text. The data are expressed as mean ± *SD* for at least 3 individual experiments. The data were analyzed by one‐way ANOVA with the Tukey HSD post hoc test. Data labeled with different symbols represent a significant difference within groups at *p* < .05

The antioxidation activity in different fractions of Annona fruit is shown in Figure 4a. The methanol extract of skin had the highest Fe^3+^ reducing activity, which was equivalent to 1739 ± 28 μM Fe^2+^/mg dry weight. The water extract of skin contained a significantly lower amount of Fe^3+^ activity (558 ± 58 μM Fe^2+^/mg dry weight) and the chloroform extract of skin contained the least amount of Fe^3+^ reducing activity (167 ± 7 μM Fe^2+^/mg dry weight).

In the pulp fraction of Annona fruit, the methanol extract had the highest Fe^3+^ reducing activity, which was equivalent to 623 ± 33 μM Fe^2+^/mg dry weight. The water and chloroform extracts contained similar antioxidation activity (120–190 μM Fe^2+^/mg dry weight) which was significantly lower than that of methanol extract. In seeds of Annona fruit, the methanol extract had the highest Fe^3+^ reducing activity (393 ± 15 μM Fe^2+^/mg dry weight). Similar to pulp extracts, the water and chloroform extracts of seeds contained similar antioxidation activity (40–60 μM Fe^2+^/mg dry weight), which was significantly lower than that of methanol extract.

The total antioxidation activity in the skin, pulp, and seed fractions of *Annona cherimola* was also determined using DPPH assay, and the data are presented in Figure 4b. Similar to the FRAP assay, the data showed that the methanol extracts of skin exhibited highest antioxidation activity (7,654 ± 35 μM Tolox/g extract) followed by pulp (2,947 ± 177 μM Tolox/g extract) whereas seeds exhibited very little antioxidation activity (66.64 ± 0.49 μM Tolox/g extract). The water extract of skin also exhibited the highest antioxidation activity (3,939 ± 62 μM Tolox/g extract); however, water extract of seeds (28 ± 5.40 μM Tolox/g extract) and pulp (37.12 ± 0.75 μM Tolox/g extract) has significantly lower antioxidation activity then that of methanol extracts. The chloroform extracts of skin, pulp, and seeds exhibited very little antioxidation activity, ranging from 6 to 40 μM Tolox/g extract.

### 3.4 Effect of annona skin extract on ramos‐1 lymphoma cell proliferation

**Figure 5:**
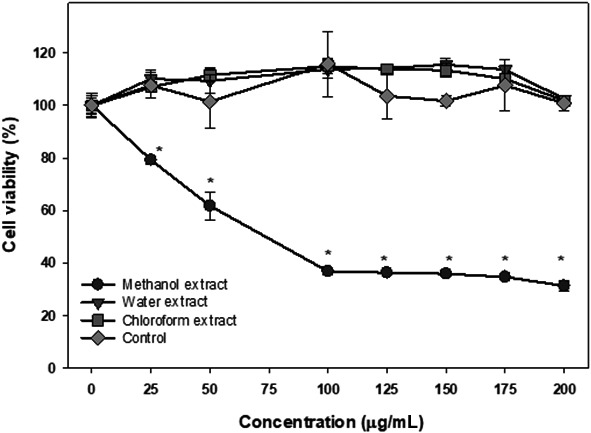
The cytotoxic effects of skin extracts on Ramos‐1 lymphoma cells. The cytotoxic effects of methanol, water, and chloroform extracts of Annona fruit's skin were determine by WST‐1 assay as described in the text. The data are expressed as mean ± *SD* for at least 3 individual experiments. The data were analyzed by ANOVA with the Tukey HSD post hoc test. Data with “*” represent a significant difference between control and treated cells with *p* < .05

The effect of different extracts of the skin of the Annona fruit was investigated on lymphoma cells and data are shown in Figure 5. The water and chloroform extracts of skin did not produce any significant antiproliferative effect on Ramos lymphoma cells. The methanol extract significantly inhibited cell proliferation at concentrations as low as 25 μg/ml of dry extract (*p* < .05) and showed a dose‐dependent effect up to 75 μg/ml of dry extract where almost 60% cells died. Increasing the concentration of Annona skin extract over 75 μg/ml did not increase the inhibitory effect**.**

### 3.5 Effect of annona pulp extract on ramos‐1 lymphoma cell proliferation

**Figure 6:**
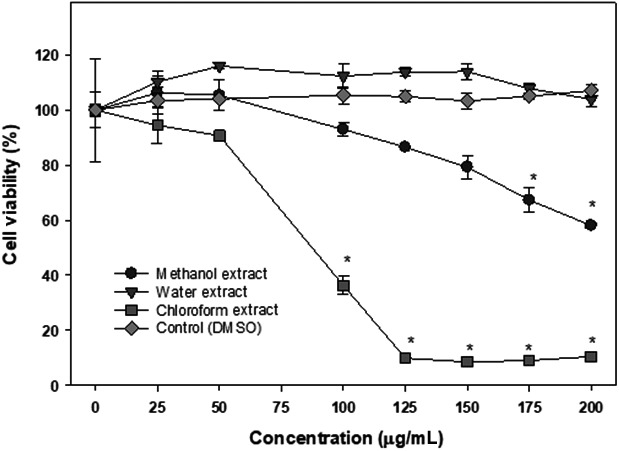
The cytotoxic effects of pulp extracts on Ramos‐1 lymphoma cells. The cytotoxic effects of methanol, water, and chloroform extracts of Annona fruit's pulp were determine by WST‐1 assay as described in the text. The data are expressed as mean ± *SD* for at least 3 individual experiments. The data were analyzed by one‐way ANOVA with the Tukey HSD post hoc test. Data with “*” represent a significant difference between control and treated cells with *p* < .05

The effect of different extracts of the pulp of the Annona fruit on lymphoma cells are shown in Figure 6. The methanol extract significantly inhibited cell proliferation in a dose ‐dependent manner with a maximum of 30% at the highest concentration at 200 μg/ml of dry extract. The chloroform extract of pulp was extremely effective in carrying out its anticancer activity peaking at around 125 μg/ml where, significantly, 90%–95% cells died (*p* < .05). The water extract from pulp had no effect on lymphoma cells.

### 3.6 Effect of annona seed extract on ramos‐1 lymphoma cell proliferation

**Figure 7:**
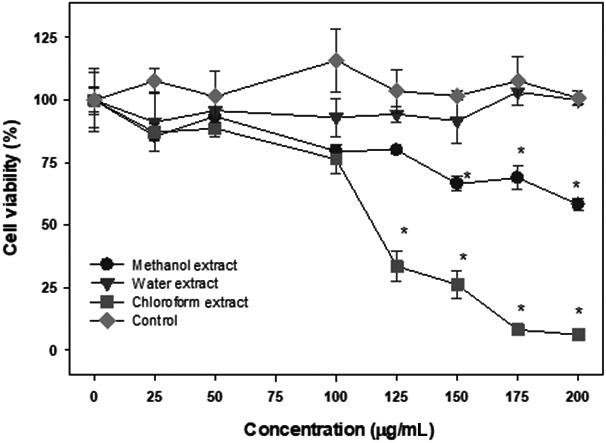
The cytotoxic effects of seeds extracts on Ramos‐1 lymphoma cells. The cytotoxic effects of methanol, water, and chloroform extracts of Annona fruit's seeds were determine by WST‐1 assay as described in the text. The data are expressed as mean ± *SD* for at least 3 individual experiments. The data were analyzed by ANOVA with the Tukey HSD post hoc test. Data with “*” represent a significant difference between control and treated cells with *p* < .05

The effect of different extracts of the seeds of the Annona fruit on lymphoma cells are shown in Figure 7. The water extract had no effect on lymphoma cell proliferation. The methanol extract had a modest effect and inhibited cell proliferation in a dose‐dependent manner causing an inhibition by 40% at 200 μg/ml (*p* < .05). The chloroform extract of the Annona fruit exhibited strongest anticancer activity. It inhibited cell proliferation in a dose‐dependent manner and caused complete cell death at the highest concentration (*p* < .05).

### 3.7 Determination of total fatty acids in the chloroform extracts of annona fruits fractions

**Figure 8:**
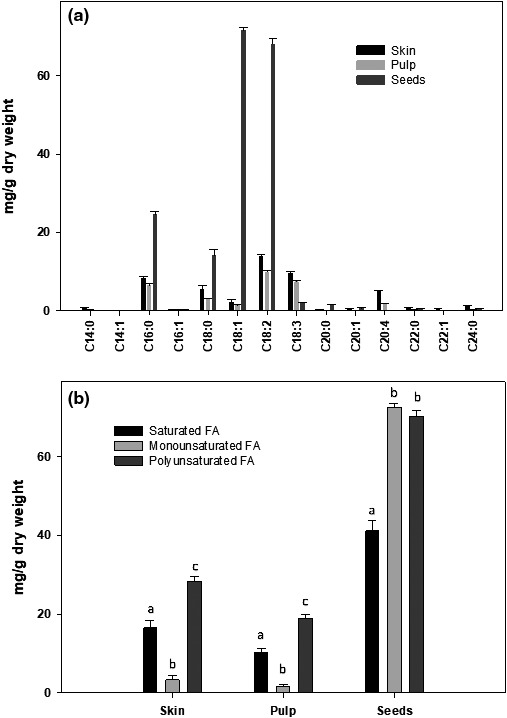
Fatty acid composition of Annona seeds. Fatty acid composition in the chloroform extracts of skin, pulp, and seeds fractions was determined using a gas chromatography system as described in the text and reported as (a) concentration of individual fatty acids and (b) concentration of fatty acids in different subclasses. The data are expressed as mean ± *SD* for at least 3 individual experiments. The data were analyzed by ANOVA with the Tukey HSD post hoc test. Data labeled with different symbols represent a significant difference within groups at *p* < .05

As indicated above, the chloroform extracts of pulp and of seeds had the strongest anticancer effects on lymphoma cells. The chloroform extracts contained nonpolar compounds, mostly fats. We, therefore, analyzed fatty acid composition of Annona fractions. The results of the fatty acid analysis are shown in Figure 8a and b. As expected, the data indicate that skin and pulp had considerably less fatty acids (35 – 60 mg/g dry weight) than that of seeds (~200 mg/g of dry weight). The skin and pulp mostly contained saturated (C16:0) and polyunsaturated (C18:2) fatty acids. The seeds contained the highest amounts of fatty acids with equal amounts of mono (C18:1) and polyunsaturated (C18:2; C18:3) fatty acids (70–75 mg/g dry weight) whereas it contained lesser amounts of saturated (C16:0) fatty acids (40 – 45 mg/g dry weight). The long chain polyunsaturated omega‐3 class of fatty acids was not detected in any fraction of Annona fruit.

## 4 DISCUSSION

In this study, we investigated the effect of various extracts of *Annona cherimola* prepared in the laboratory on the proliferation of lymphoma cancer cells. Lymphoma is a cancer that begins in the lymph nodes, which are involved in the proliferation and maturation of immune cells, lymphocytes. In lymphoma, lymphocytes mutate and grow out of control. During the present investigation we studied non‐Hodgkin lymphoma using Ramos‐1 cancer cell lines. Ramos‐1 cells were isolated from a 3‐year‐old Caucasian male patient with Burkitt's lymphoma involving abnormal proliferation of B lymphocytes (Wu, Martin, Carrington, & KewalRamani, 2004). As mentioned in the introduction section, for most lymphomas, the standard treatment is a combination of chemotherapy and radiotherapy (Banerjee, 2011; Stathis, 2016). However, the combination therapy used in the treatment of lymphoma has a number of adverse side effects and can effect cardiovascular, gastrointestinal, hematological, urogenital, respiratory, skin, renal, neurological, and reproductive systems (Hodgson, 2015).

Antioxidants and phytochemicals present in vegetables and fruits may inhibit the progression of the tumor through the effect on improving the immune system function, reducing oxidative stress by antioxidant pathways, and modulation of detoxification enzymes (Colitti, Stefanon, Gabai, Gelain, & Bonsembiante, 2019). Annona fruits have powerful antioxidation activity and also possess anticancer properties because of their polyphenolic content (Yajid, Ab Rahman, Wong, & Wan Zain, 2018). Phenolic compounds, or polyphenols, are widely distributed group of chemicals in the plant kingdom. A rough estimate suggest that more than 8,000 phenolic structures are currently known (Harborne & Williams, 2000). Phenolic compounds can be simple molecules, such as phenolic acids, or they can be highly polymerized and form complex compounds, such as proanthocyanidins (tannins), and commonly present in many foods (fruits, vegetables, and cereal grains) and beverages (wine, beer, teas) (Manach, Scalbert, Morand, Rémésy, & Jiménez, 2004).

Among all fractions, methanol extracts of skin contained the highest concentration of polyphenols, flavonoids, and tannins than other extracts of skin, pulp, and seeds. The polyphenols in skin not only contribute to apparent fruit's color but also play an important role to prevent attacks from microbes such as bacteria, algae, and fungus (Lattanzio, Veronica, Lattanzio, & Cardinali, 2006). In most cases, skin is not edible because of its bitter taste and also presence of tannins which can be poisonous (Seeram et al., 2006); however, tannins present in the skin of Annona fruits have been reported to be nontoxic (Brat et al., 2006) whereas seeds are not edible as they contain toxic tannins. It is interesting to note that the total amount of polyphenols are about two times higher in pulp as it contained about 75% of the fruit mass than that of skin which only represents about 13% total mass. Recent studies have identified a total of 21 phenolic compounds in the cherimoya pulp including quince acid, caffeic acid, coumaric acid, procyanidine, proanthocyanidine, catechins, and quercetins (García‐Salas, Gómez‐Caravaca, Morales‐Soto, Segura‐Carretero, & Fernández‐Gutiérrez, 2015). Other studies have also reported the presence of a different range of 20 compounds in unspecified cherimoya pulp, mainly hydroxycinnamic and hydroxybenzoic acids (Spínola, Pinto, & Castilho, 2015). The types and concentrations of these compounds in Annona depend on many factors including type of plant or cultivar, genetic factors, and maturity stage (Albuquerque et al., 2016). During the present investigation, we did not characterize or identify the nature the phenolic compounds present in Annona fruits. However, we investigated total flavonoid contents in Annona fractions. Flavonoids is a diverse group containing various subclasses including flavanol, flavanones, flavones, isoflavones, flavanols, and anthocyanidins (Panche, Diwan, & Chandra, 2016). It is difficult to measure total flavonoids because of their variable reactivity with AlCl_3._ We used two different methods based on their reactivity with NaNO_2_ as previously reported (Pekal & Pyrzynska, 2014; Struchkov et al., 2018). Our data indicate that amount of flavonoids were significantly lower in methanol and chloroform extracts when assayed without NaNO_2_. This could be due to fact that this method mostly measures flavonol and flavone subclasses (Pekal & Pyrzynska, 2014) whereas the assay with NaNO_2_ measures flavan‐3‐ols, flavanones, and isoflavones, and anthocyandines (Pekal & Pyrzynska, 2014; Struchkov et al., 2018). It is suggested that current assays for flavonoids unequivocally reflects total flavonoids content in the nature products because of variability in reactivity of different flavonoids with AlCl_3_ (Struchkov et al., 2018). Interestingly, water extracts of all fractions resulted in similar flavonoid contents using both procedures. This suggests that perhaps both assays were able to detect a particular class of water‐soluble flavonoids. Further work is required to identify the flavonoids detected by different procedures.

Evidence indicates that Annona fruit pulp is rich in antioxidant compounds (Loizzo et al., 2012). Our data showed that methanol extracts from skin and from pulp also showed the highest antioxidation activities. The high antioxidation activity in the methanol fractions is consistent with the highest phenolic contents in this fraction. It is generally known that phenolic compounds are typically responsible for the antioxidant activity in plant fruits. The antioxidant activity of polyphenols is attributed to their redox properties, that is, adsorbing and neutralizing free radicals, quenching singlet and triplet oxygen, and decomposing peroxides. Medicinal plant tissues are commonly rich in phenolic compounds such as flavonoids, phenolic acids, stilbenes, tannins, coumarins, lignans, and lignins. However, these compounds have other biological effects in addition to their antioxidant activity including their effect on different types of cancer (Rasouli, Farzaei, & Khodarahmi, 2017).

Our data indicate that methanol extracts from skin, pulp, and seeds have a moderate effect; however, chloroform extracts from pulp and from seeds are more effective in killing lymphoma cancer cells. The methanol extracts likely contain polar polyphenols of low to moderate molecular weights whereas the chloroform extracts likely contain high‐molecular weight nonpolar polyphenols or water‐insoluble lipid compounds. Since the chloroform fraction exhibited strong anticancer properties, we further evaluated this fraction. We determined the fatty acid profiles in the chloroform extracts to see if any differences in fatty acid distribution among these fractions can be correlated with their anticancer activity. As expected, seeds have the highest amount and the pulp has the lowest amount of fatty acids. The unsaturated fatty acids were more prevalant than the saturated fatty acids in all fractions. The skin and pulp contained mostly saturated (16:0) and polyunsaturated (18:2, 18:3) fatty acids but very few monounsaturated fatty acids (18:1). In contrast, the seeds contained equal amounts of monounsaturated and polyunsaturated fatty acids. It is apparent from data that differences in fatty acid profiles cannot be correlated with their anticancer activity.

It is known that nutrients involved in antioxidant activities may protect against the development of NHL because lower intakes of antioxidants have been linked to a compromised immune system (Brambilla, Matsumoto, Araujo, & McKinlay, 2009). Several studies have shown that a reduced risk of NHL is associated with a high vegetable and fruit intake (Thompson et al., 2010; Zhang et al., 2000). In one study about 600 patients with NHL were surveyed for their fruits and vegetables consumption. The study found that high intakes of vegetables and fruits, vegetables, green leafy vegetables, and citrus fruits gave 27%–42% lower risks of death in patients with NHL overall compared to low‐intake groups, after adjustment for demographic and clinical prognostic factors. Patients with follicular lymphoma who had high intakes of vegetables and green leafy vegetables experienced a 73% reduced risk of death (Han et al., 2010). Furthermore, a prospective study of older Iowan women found an overall inverse association for intakes of both fruits and vegetables with risk of NHL (Thompson et al., 2010). They also found that dietary intake of antioxidants including carotenoids, vitamin C, proanthocyanidins, and manganese also exhibited an inverse association with NHL risk. The associations were strongest for yellow/orange and cruciferous vegetables; for fruits, the strongest association was for apple juice/cider; and for the carotenoids, the strongest association was for α‐carotene. Interestingly, foods with strong antioxidant properties, including whole grains, nuts, chocolate, tea, and red wine, were not associated with NHL risk. These observations suggest that in addition to antioxidation properties, the structure of phenolic compounds also play some important role in inhibiting cancer growth. Our data also show that the methanol fractions of skin, pulp, and seeds contained the highest amounts of polyphenols, flavonoids, tannins, and antioxidation activity but these extracts have moderate anticancer activity compared with chloroform extracts that contained low concentration of polyphenol and showed low levels of antioxidation activity but exhibited high antiproliferative activity. Our data suggest that Annona fruit in addition to its phenolic compounds also contained some other highly nonpolar compounds which also contribute to its anticancer activity. Interestingly, a cyclic heptapeptide was isolated from Annona seeds that also exhibited anticancer activity in KB cells (Wélé et al., 2004). The pulp from Annona fruits represents about 75% mass of the fruit and is the edible part of the fruit whereas the skin and seeds are not edible. Interestingly, both methanol and chloroform extracts of pulp exhibited anticancer activities. Therefore, regular consumption of Annona fruit may be effective to prevent or treat lymphoma. During the present investigation we have not determined the phytochemical profile of different extracts of the Annona fruit fraction. Further studies are required to determine the phytochemical profile of Annona fruit and to study their anticancer effects in an animal model.

In conclusion, our data indicate that both methanol and chloroform extracts of pulp exhibited anticancer activities and that regular consumption of pulp from Annona fruit may be an effective way to prevent or treat lymphoma. Further research is needed to identify and characterize the active compounds from the Annona fruits for potential therapeutic use and for a better understanding of the underlying mechanism. Although the present study is focused only on *Annona cherimola* species, there is a possibility that other species belonging to *Annona* genus may have even more potent antioxidation and anticancer activities as environmental conditions may influence the composition of active compounds. Thus, further investigation is required to test other species of Annona fruits in order to find the species with optimal health benefits.

## CONFLICT OF INTEREST

All authors declare no conflict of interest.

## ETHICAL APPROVAL

No human subjects or vertebrate animals were used in this study.
